# Fueling immunity: the synergy of natural products and exercise for optimal health

**DOI:** 10.3389/fphar.2025.1582540

**Published:** 2025-07-31

**Authors:** Fuzhen Li, Wei Zhou, Jun Wang

**Affiliations:** ^1^ Qingdao University of Technology, Qingdao, Shandong, China; ^2^ China University of Petroleum (East China), Qingdao, Shandong, China

**Keywords:** natural products, exercise, inflammation, immunity, effect

## Abstract

Moderate exercise has been associated with improved immune function and a reduced risk of inflammatory conditions and infections. Some evidence also suggests a potential role in reducing cancer risk. In contrast, excessive physical activity can suppress the immune system and increase the risk of inflammatory and allergic conditions. The vulnerability to infections associated with overexertion is linked to elevated levels of immunosuppressive factors, such as adrenocortical hormones and anti-inflammatory cytokines. These changes reduce the number and activity of natural killer (NK) cells and T cells and decrease IgA levels in saliva. Consequently, athletes engaged in high-intensity training may face a greater risk of compromised immune function. In the sports nutrition market, a range of natural products is available, but many lack clear evidence of effectiveness and are marketed with misleading claims, leading to consumer confusion. The efficacy of food components discussed in this article warrants further investigation due to differing opinions in research. Additionally, the effectiveness of these components may vary by gender, differences, and method of consumption. Therefore, future research is needed to determine optimal intake methods and timing of these products based on their intended use and physiological changes resulting from exercise. This review explores the effects of natural products combined with exercise on maintenance and reduction.

## 1 Introduction

The interplay between diet, physical activity, and immune function is a burgeoning area of research that highlights the significant role of natural products and exercise in promoting health and wellbeing. Natural products, defined as those that provide health benefits beyond basic nutrition, have gained attention for their potential to enhance immune responses and reduce inflammation. Concurrently, regular exercise is recognized for its ability to bolster immune function and mitigate the risk of chronic diseases, making it a critical component of a healthy lifestyle. As our understanding of the immune system deepens, it becomes clear that both moderate physical activity and specific dietary components can work synergistically to optimize immune health. Moderate exercise is known to stimulate immune cell activity, improve circulation, and enhance the body’s ability to respond to infections (Nieman and Wentz, 2019). In contrast, excessive physical exertion can lead to immunosuppression, highlighting the importance of balance in exercise routines (Nieman and Wentz, 2019; Simpson et al., 2020). This duality emphasizes the necessity for athletes and individuals engaged in high levels of physical activity to pay particular attention to their nutritional intake to support immune resilience. Natural products encompass a diverse range of dietary components, including probiotics, prebiotics, omega-3 fatty acids, antioxidants, and various bioactive compounds (Vignesh et al., 2024). These natural products possess unique properties that can support immune function, combat inflammation, and enhance overall health (Vignesh et al., 2024). For instance, probiotics help maintain a balanced gut microbiota, which is crucial for optimal immune function (Hemarajata and Versalovic, 2013), while omega-3 fatty acids are known for their anti-inflammatory effects (Zivkovic et al., 2011). Antioxidants, found in fruits, vegetables, and whole grains, can protect against oxidative stress, a key factor in the development of chronic inflammatory conditions (Rahaman et al., 2023). Moreover, emerging research suggests that the effectiveness of these natural products may vary based on individual characteristics such as age, sex, and genetic predispositions. This variability highlights the complexity of nutritional science and emphasizes the importance of personalized approaches to diet and exercise. Understanding how different natural products and exercise modalities interact can lead to tailored health strategies that maximize benefits for diverse populations. This article aims to explore the beneficial effects of natural products in conjunction with exercise on immune maintenance and inflammation reduction. By examining the mechanisms through which these interventions operate, as well as their potential synergies, we seek to provide a comprehensive overview of how lifestyle modifications can enhance immune resilience and promote overall health. We will delve into recent studies that illustrate the impact of specific natural products and exercise types on immune markers and inflammatory responses, shedding light on the pathways involved. Through this review, we hope to contribute to the growing body of evidence supporting the integration of natural products and exercise as a strategy for improving immune function and reducing chronic inflammation.

### 1.1 Methodology and literature search strategy

To ensure a comprehensive and unbiased review, we conducted a structured literature search across PubMed, Scopus, and Google Scholar using a combination of Medical Subject Headings (MeSH) terms and free-text keywords, including “natural products,” “exercise,” “immune function,” “synergy,” “inflammation,” “immunomodulation,” and “physical activity.” The search was limited to articles published in English from January 2000 to April 2025. We selected studies based on their relevance to the interaction between natural products and exercise and their impact on immune response or inflammation. Inclusion criteria encompassed peer-reviewed original research or systematic reviews involving human participants or animal models relevant to exercise and immunity, with a focus on one or more natural compounds reported to have immunological effects. We excluded non-English articles, case reports, editorials, and studies not directly addressing the synergy between natural products and exercise. References were also manually screened for relevance, and duplicate studies were removed.

## 2 Overview of the immune system

**FIGURE 1:**
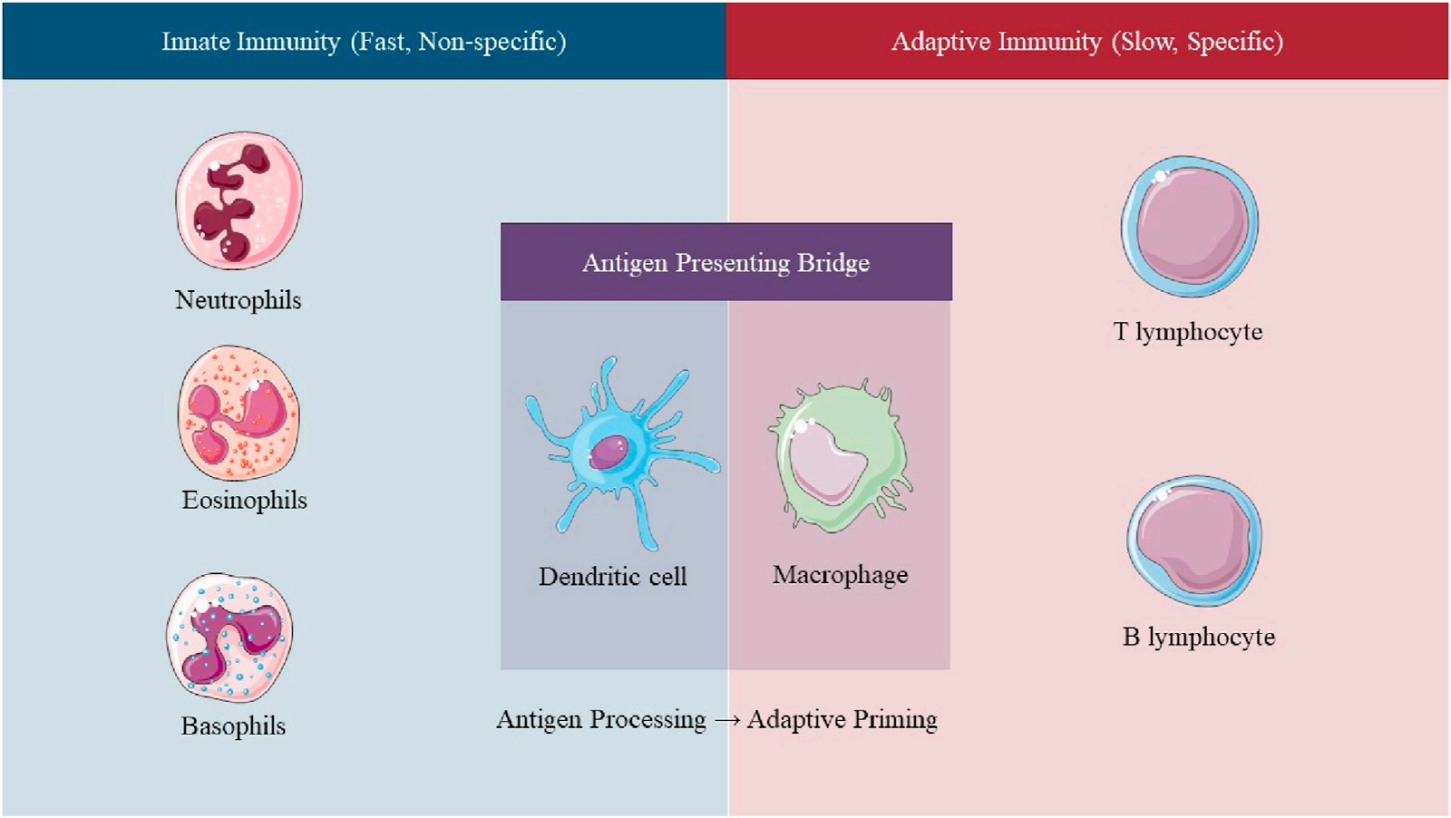
Schematic representation of the innate and adaptive immune responses. The innate system acts rapidly and non-specifically, while the adaptive system provides delayed but specific immunity through T and B lymphocytes. The figure highlights their temporal dynamics and complementary roles in host defense.

The immune system is a highly complex network that functions optimally in a nutrient-rich environment, which is crucial for immune cells’ activation, differentiation, and interplay. It comprises two major branches: innate immunity and adaptive immunity (Noor et al., 2021; Marshall et al., 2018; Wang et al., 2024) (Figure 1). Innate immunity represents the first line of defense and responds rapidly and non-specifically to invading pathogens (Marshall et al., 2018). Key components include macrophages, dendritic cells (DCs), NK cells, and pattern recognition receptors such as Toll-like receptors (TLRs) (Marshall et al., 2018; Fitzgerald and Kagan, 2020). Macrophages engulf pathogens via phagocytosis and release pro-inflammatory cytokines such as TNF-α to recruit other immune cells, including neutrophils and eosinophils (Hirayama et al., 2017; Chen et al., 2023). They also generate nitric oxide (NO) through inducible synthase (iNOS) to exert antimicrobial effects. As essential pattern recognition receptors (PRRs), TLRs detect conserved molecular patterns (PAMPs) on pathogens. For example, activation of TLR2 or TLR4 initiates intracellular signaling cascades (e.g., NF-κB, MAPK) that upregulate inflammatory gene expression and enhance innate immune responses (Fitzgerald and Kagan, 2020).

NK cells play a critical cytotoxic role in antiviral and anticancer immunity. They detect and eliminate infected or transformed cells by releasing cytolytic granules containing perforin and granzymes, which induce apoptosis. NK cells also secrete interferon-gamma (IFN-γ) to activate macrophages and enhance their antimicrobial functions (Wang et al., 2024). DCs act as antigen-presenting cells (APCs) that bridge innate and adaptive immunity by capturing antigens and presenting them to T cells.

Adaptive immunity is antigen-specific and forms long-lasting immunological memory. It involves T and B lymphocytes, which orchestrate targeted immune responses (Wang et al., 2024). B cells produce antibodies that neutralize pathogens and facilitate phagocytosis while activating the complement cascade. CD8^+^ cytotoxic T lymphocytes (CTLs) eliminate infected or malignant cells via perforin/granzyme pathways and Fas–FasL-mediated apoptosis (Raskov et al., 2021). CD4^+^ helper T cells (TH cells) regulate immune responses, supporting both humoral and cellular immunity by producing cytokines and aiding other immune cells (Raskov et al., 2021; Sun et al., 2023). TH cells differentiate into various subsets such as TH1 (promote cell-mediated immunity via IFN-γ and TNF-α), TH2 (stimulate B cell activity and antibody production through IL-4 and IL-10), and TH17 (enhance mucosal immunity via IL-17, IL-22, and IL-6) (Sun et al., 2023). Immune function is influenced by age, sex, nutritional status, and underlying health conditions, all of which can alter susceptibility to infection.

## 3 The role of exercise in immune function and inflammation

The relationship between exercise and immune function is often described using the “J-shaped” curve model, initially proposed by Nieman in the context of upper respiratory tract infections (Nieman, 1994). According to this model, moderate and regular physical activity enhances immune surveillance and lowers the risk of infections by stimulating immune components such as NK cells and T lymphocytes (Nieman, 1994). In contrast, excessive or high-intensity exercise (HIIT), particularly when coupled with insufficient recovery, may lead to a transient immunosuppressive state. This suppression is associated with elevated cortisol and anti-inflammatory cytokines, resulting in reduced immune responsiveness and increased susceptibility to infections (Syed et al., 2024). Furthermore, exercise modulates levels of pro-inflammatory cytokines (Lee et al., 2023). While moderate activity tends to reduce these markers, intense training may initially elevate them but potentially improve inflammatory balance over time.

## 4 The role of natural products in immune function and inflammation

There are numerous definitions for natural products, which include natural products promoted with health benefits, items that offer positive physiological effects beyond just providing essential nutrients, and natural substances intended for daily consumption that can influence or regulate bodily systems when ingested (Essa et al., 2023). The ingredients in natural products, influenced by technological factors, can aid in disease prevention and enhance the performance and wellbeing of consumers, extending beyond their nutritional functions. This effect can apply to the general population or specific groups defined by age or genetic variations.

Natural products provide health benefits that extend beyond basic nutrition, influencing bodily systems and potentially aiding in disease prevention (Essa et al., 2023). Various food components, such as probiotics, prebiotics, omega-3 fatty acids, and antioxidants, have been highlighted for their positive effects on the immune system and inflammation (Essa et al., 2023). Probiotics help maintain gut microbiota balance, enhance gut barrier function, and reduce systemic inflammation by modulating immune responses (Filidou et al., 2024). Prebiotics promote the growth of beneficial gut bacteria, strengthening the gut barrier and reducing the likelihood of harmful substances entering the bloodstream, thereby mitigating inflammation (Ji et al., 2023). Omega-3s, found in fish oil, are known to reduce inflammation by modulating cytokine production and enhancing immune cell function (Banaszak et al., 2024). Many natural products are rich in antioxidants, which combat oxidative stress—a key factor in the inflammatory response. This helps protect tissues and supports overall immune health.

## 5 Synergistic effects of natural products and exercise on immunity and inflammation

### 5.1 Probiotics

Probiotics—especially strains such as *Lactobacillus* plantarum, *Lactobacillus* rhamnosus, and Bifidobacterium bifidum—demonstrate immunomodulatory and anti-inflammatory properties. These beneficial effects are achieved by reshaping gut microbiota, enhancing gut barrier function, reducing pathogen translocation, and producing short-chain fatty acids (SCFAs) (Mazziotta et al., 2023). These SCFAs modulate systemic immunity through the gut–brain and gut–liver axes by downregulating inflammatory pathways (Mazziotta et al., 2023; Quigley et al., 2020). This enlightening information about the potential of probiotics can inform and empower individuals about the benefits of incorporating probiotics into their diet.

Physical activity, particularly regular exercise such as aerobic training, high-intensity interval training (HIIT), Pilates, and circuit training, plays a significant role in reducing pro-inflammatory cytokines (e.g., TNF-α, IL-6), increasing anti-inflammatory mediators like IL-10, improving antioxidant capacity, and enhancing BDNF and immune surveillance (Quigley et al., 2020).

This highlights the crucial role of exercise in enhancing immune function and promoting overall health. This inspiring revelation can motivate individuals to incorporate physical activity into their daily routine for a healthier life. Recent studies have uncovered the potential of probiotics and exercise to collaborate, unveiling synergistic, additive, or occasionally null outcomes, depending on the intervention parameters (Table 1). This emerging field of research is particularly intriguing, as it offers new insights into the potential of combined interventions. For instance, in models of non-alcoholic fatty liver disease (NAFLD), combinations of HIIT with L. rhamnosus consistently yielded synergistic suppression of key inflammatory mediators (TLR4, MYD88, NF-κB) and improved lipid markers (Eyni Gandomani and Reisi, 2020; Kayacan et al., 2022; Rasheh and Ahmadi, 2021). A study on postnatal women showed that daily probiotic supplementation combined with Pilates reduced inflammatory markers (IL-6, TNF-α), leptin levels, and body fat significantly more than either intervention alone, supporting a synergistic metabolic and immunological effect (Mazinani et al., 2021). In diabetic rats, co-intervention with probiotics and treadmill running improved antioxidant defenses (↑TAC, ↑ superoxide dismutase (SOD)) and reduced glucose levels, again indicating synergism (Ibrahim et al., 2018b). Similarly, probiotic-fermented soy milk paired with graded treadmill exercise enhanced splenocyte proliferation and reduced TNF-α beyond single interventions, reflecting a gut–immune axis synergy (Appukutty et al., 2015).

**TABLE 1:** Impact of probiotic, prebiotic and exercise on immunity and inflammation.

Natural products	Dose/Duration	Exercise	Exercise protocol	Disease/Condition	Mechanism	Synergistic/Additive	Ref.
*Lactobacillus* rhamnosus		HIIT	5 sessions/week for 5 weeks	NAFLD	↓ TLR4 and MYD88 gene expression in gut tissue → reduction in inflammation	Synergistic	Mazinani et al. (2021)
Probiotic	1 capsule per day	Pilates	2×/week for 8 weeks; 10 min warm-up, 30 min main, 10 min cool-down	Postnatal women	↓ Harmful gut bacteria, ↓ leptin, ↓ IL-6, ↓ TNF-α, ↓ body fat, WHR	Synergistic	Hyun et al. (2020)
Probiotic (VSL#3 mixture)	20 mg probiotic VSL#3	Swimming	5 weeks moderate swimming (1 h/day, 5 days/week) + 1 week exhaustive (3 × 150 min/day with rest intervals, 5 days/week)	Exercise-induced oxidative stress	↓ Oxidative stress; ↑ SOD, catalase, GSH in organs; colon morphology altered (↓ crypt depth and mucosa thickness)	Additive	Ünsal et al. (2018)
Multi-strain Probiotics	3 × 10^10^ CFU twice daily for 12 weeks	Circuit Training	3×/week; weeks 1–8: 2 circuits/session; weeks 9–12: 3 circuits/session	Sedentary lifestyle, low muscular strength/power	↑ IL-10 in probiotic and CT groups; ↑ isokinetic strength and power in CT and CTP groups	Additive	Ibrahim et al. (2018a)
Multi-strain Probiotics	Twice daily for 12 weeks	Circuit Training	3×/week for 12 weeks; 10 resistance exercises/circuit, work:rest ratio 1:2, progressive load	healthy young males with a sedentary lifestyle	Circuit training ↑ total leukocytes, lymphocytes, T cells; Probiotics had no significant immune effect	No effect	Ibrahim et al. (2018b)
Soy milk fermented with *Lactobacillus* plantarum LAB12	Oral gavage for 42 days	Treadmill	Graded treadmill running	Immune modulation in trained condition	↑ Splenocyte proliferation; ↓ TNF-α production in LAB12 and LAB12+Exercise groups	Synergistic	Appukutty et al. (2015)
Probiotic	2 gr dissolved in 30 mL water/rat/day for 4 weeks	Treadmill	4 weeks	Type 2 Diabetes	↑ TAC, ↑ SOD, ↓ Glucose levels	Synergistic	Maherinia et al. (2020)
Probiotic mix (L. rhamnosus, L. paracasei, L. acidophilus, B. lactis)	6 × 10^8^ CFU daily for 8 weeks (oral capsule)	Treadmill	8 weeks	Oxidative stress (exercise-induced)	↑ Total thiol (TT) in exercise, ↓ Dynamic disulfide (DD) with probiotics + exercise → ↓ oxidative damage	Synergistic	Kayacan et al. (2022)
Probiotic VSL#3	Given in drinking water during 5 weeks	Moderate swimming + Intensive swim	5 weeks	Rats with Gut barrier disruption, oxidative/inflammatory stress	↓ Zonulin, ↓ MDA and protein carbonyl, modulated IL-6, TNF-α, IL-10	Synergistic during moderate exercise	Ünsal et al. (2017)
Probiotic	5 weeks and 5 days	HIIT	5 sessions/week for 5 weeks	NAFLD	↓ IL-10, ↓ IFN-γ gene expression in gut tissue	Synergistic	Rasheh and Ahmadi (2021)
*Lactobacillus* rhamnosus GG	10^9^ CFU/mL daily for 5 weeks	HIIT	5x/week for 5 weeks	Hepatic steatosis	↓ NF-κβ and ↓ CXCL2	Synergistic	Mohammadi et al. (2022)
Lacticaseibacillus rhamnosus GG (LGG)	10^7^ CFU/mL, gavage, 5x/week for 5 weeks	HIIT	5x/week for 5 weeks	Tetracycline-induced hepatic steatosis	LGG + HIIT ↓ LDL, cho, TG; ↑HDL and SOD; ↓ALP, AST, ALT; ↓hepatic lipid droplets	Synergistic	Aghaei et al. (2023)
Lycium barbarum polysaccharides (LBP)	50 mg/kg daily/8 weeks	Treadmill	5 days/week for 8 weeks	NAFLD	↑ Gut microbiota diversity; ↑ SCFA; ↑ZO-1, occludin; ↓ LPS/TLR4/NF-κB signaling; ↓ hepatic inflammation and intestinal permeability	Synergistic	Gao et al. (2021)

In contrast, a study combining VSL#3 with swimming showed enhanced antioxidant enzyme activity (↑SOD, ↑CAT, ↑GSH), but the effect was additive, not synergistic—each intervention contributed independently (Ünsal et al., 2018). Circuit training combined with multi-strain probiotics in sedentary adults improved IL-10 and muscle performance, but the overall interaction was also additive, as no synergistic immune benefit was observed (Ibrahim et al., 2018a).

Notably, in healthy young males, probiotics had no significant immune effect when combined with circuit training, despite improvements in leukocyte counts from exercise alone, indicating a non-synergistic or null effect (Hyun et al., 2020). Moreover, the interaction outcome depends on multiple moderators: the strain and form of probiotics (e.g., fermented food vs. capsule), dosage, duration, exercise intensity, and host condition. For example, moderate exercise intensity is often associated with optimal immunological synergy, whereas high-intensity regimens may impair gut barrier integrity and override probiotic benefits (Ünsal et al., 2017). In some studies, multi-strain formulations—particularly those producing SCFAs—have shown more pronounced synergistic effects than single strains (Ibrahim et al., 2018a; Ünsal et al., 2017; Gao et al., 2021). However, the benefits remain complementary or additive in other contexts, underscoring the need for precise characterization in describing these interactions.

### 5.2 Prebiotics

Regular aerobic exercise, combined with prebiotic supplementation such as Lycium barbarum polysaccharides (LBP), has demonstrated synergistic effects in enhancing gut microbiota diversity and composition in animal models of NAFLD (Gao et al., 2021).

This combination significantly increases the relative abundance of beneficial bacterial phyla, particularly Bacteroidetes, while reducing Proteobacteria and the Firmicutes/Bacteroidetes ratio—markers typically associated with dysbiosis and metabolic dysfunction. This improved microbial profile leads to elevated SCFAs production, which upregulate the expression of tight junction proteins such as ZO-1 and occludin, thereby enhancing gut barrier integrity (Gao et al., 2021).

Improved barrier function is associated with reduced intestinal permeability and lower lipopolysaccharides (LPS) translocation into circulation. Consequently, systemic inflammation is mitigated by downregulating the LPS/TLR4/NF-κB signaling pathway, a key driver of hepatic inflammation in NAFLD. The significant role of aerobic exercise in these physiological improvements, especially in terms of hepatic inflammation reduction and gut barrier restoration, was found to be greater in the combined intervention than LBP or aerobic exercise alone, supporting the synergistic nature of the interaction (Gao et al., 2021). This highlights the importance of aerobic exercise in the treatment of NAFLD. Aerobic exercise contributes primarily through systemic metabolic and anti-inflammatory mechanisms (Gao et al., 2021; Luo et al., 2024). At the same time, LBP exerts its effects locally within the gut by modulating microbial composition and barrier integrity, thereby highlighting the specific role of LBP in this combined strategy (Gao et al., 2021; Li et al., 2024). The convergence of these pathways highlights the potential of this combined strategy as a therapeutic approach for managing NAFLD and related metabolic disorders.

### 5.3 Omega-3 fatty acids

Several human and animal studies have explored the relationship between omega-3 polyunsaturated fatty acids (n-3 PUFAs), particularly EPA and DHA, and physical exercise. The results of these studies, which show a combined anti-inflammatory effect in some cases and an additive or neutral outcome in others, have important implications for different populations. These implications are influenced by factors such as the type of exercise, omega-3 formulation, dosage, and target population (Table 2). For instance, in obese animal models, the combination of chronic treadmill exercise with flaxseed oil supplementation (a source of ω-3 PUFAs) led to a synergistic upregulation of GPR120 and β-arrestin-2, which in turn reduced hepatic inflammation and improved insulin signaling and physical performance, indicative of clear molecular synergy (Gaspar et al., 2019). Similarly, Tartibian et al. (2011) showed that daily 1,000 mg omega-3 supplementation combined with moderate aerobic exercise significantly improved bone mineral density (BMD) and reduced inflammatory cytokines (TNF-α, IL-6) in postmenopausal women, again suggesting a synergistic interaction. Other studies support additive effects.

**TABLE 2:** Impact of omega-3 fatty acids and exercise on immunity and inflammation.

Natural products	Dose/Duration	Exercise	Exercise protocol	Disease/Condition	Mechanism	Synergistic/Additive	Ref.
n-3 PUFA (DHA-rich fish oil)	6 g/day	Walking	3x/week, 45 min at 75% HRmax	Cardiovascular disease risk, immune function, inflammation	↓Neutrophil superoxide	Additive	Hill et al. (2007)
Flaxseed oil (ω-3 PUFA)	100 µL/day for 4 weeks	Acute and chronic treadmill	60 min; 5 days/week for 4 weeks	Obesity/T2D/inflammation	↑ GPR120 (not GPR40), ↑ β-arrestin-2, ↓ hepatic inflammation, improved insulin signaling, ↑ performance	Synergistic	Gaspar et al. (2019)
Omega-3 Fatty Acids	1,000 mg/day (24 weeks)	walking/jogging	3x/wk, 65% HRmax,24 weeks	Post-menopausal women	↑ BMD, ↑ osteocalcin, ↓ TNF-α, IL-6, PGE2, ↓ CTX, ↑ estrogen,1,25 Vit D, and calcitonin; ↓ PTH	Synergistic	Tartibian et al. (2011)
Omega-3 (EPA + DHA)	3.0 g/day (12 weeks)	whole-body resistance	3x/week for 12 weeks	Aging/inflammation/sarcopenia	↑ Lean mass, ↓ % body fat, ↑ BMD, ↑ strength, ↑ functional ability; no significant changes in IL-6 or TNF-α with omega-3	No effect	Cornish et al. (2018)
Fish oil	0.2 cc/day for 8 weeks	Treadmill	5×/weeks, 8 weeks	Exercise-induced inflammation	↓ CRP, ↓ IL-17, ↓ CK	Additive	Alizadeh et al. (2014)
Omega-3 (EPA/DHA)	3,000 mg/day, 8 weeks	Resistance training (3x/week, 8 weeks)	8 weeks, 3×/wk, 50%→80% 1RM	Muscle damage/Inflammation	↓ IL-17, ↓ CRP, ↓ CK in supplement and supplement + training groups; ↑ in training-only group → anti-inflammatory and protective effect	Preventive/Additive	Hosseini et al. (2015)
Omega-3 (EPA/DHA) + High Protein	2.2 g/day Omega-3 + 1.2–1.5 g/kg protein/day	Resistance + Vibration	8 weeks; whole-body vibration + home-based resistance	Inflammaging/Aging-Related Inflammation	↓ Circulating IL-10, ↓ IL-1RA, ↓ LPS-stimulated CCL-2 (esp. in men); ↓ IL-1RA gene expression in PBMCs	Additive/Sex-specific	Haß et al. (2023)
Omega-3 (EPA/DHA)	2,000 mg/day	Aerobic Training	8 weeks, 3×/week, 50%–70% HRmax	Obesity-related Inflammation and Lipid Dysfunction	↓ CRP in S, T, ST; ↓ MDA in T, ST; lipid profile improved in T, ST	No effect	Montazer et al. (2021)
Omega-3 (EPA + DHA)	0.06 mL/g of body weight/daily for 8 weeks	Aerobic and Anaerobic Training	aerobic: 5×/week; anaerobic: 3×/week	Exercise-induced inflammation	Omega-3 effects depended on training type; IL-17 and CRP changes varie	Variable	Alizadeh et al. (2015)
Omega-3	2000 mg/day	Aerobic training	8 weeks, 3×/week	Insulin resistance/Metabolic dysfunction	↑ Adiponectin, ↓ CTRP-9 and insulin resistance in training and training + supplement groups	Additive	Shoaei Makanet et al. (2023)

Hill et al. (2007) reported that DHA-rich fish oil reduced neutrophil oxidative stress, while moderate aerobic activity preserved immune function, suggesting that both interventions act through complementary, but distinct, mechanisms. In trials examining exercise-induced inflammation, omega-3 supplementation significantly lowered CRP, IL-17, and creatine kinase (CK) levels, implying an additive or preventive effect rather than true synergy (Alizadeh et al., 2014; Hosseini et al., 2015).

Similarly, omega-3s combined with resistance or aerobic training improved adiponectin levels and lipid profiles in overweight individuals, though these effects were typically not significantly greater than training alone (Montazer et al., 2021; Shoaei Makanet et al., 2023). Interestingly, some studies in healthy individuals without overt inflammation found no additional benefit of omega-3 supplementation when combined with exercise. For instance, Cornish et al. (2018) observed improvements in lean mass and strength with resistance training alone, while omega-3 did not further reduce IL-6 or TNF-α. Likewise, in sex-specific responses, omega-3 plus protein supplementation altered some inflammatory markers only in men, suggesting context-dependent or moderated effects (Ghyasi et al., 2019). These findings suggest that combining omega-3 fatty acids and exercise may yield synergistic or additive benefits, particularly in populations with chronic inflammation, muscle injury, metabolic syndrome, or aging-related immune dysregulation. These benefits appear to operate through pathways such as GPR120 activation, NF-κB suppression, and reductions in IL-17, CRP, and CK levels. However, omega-3s may exert minimal or no synergistic impact in healthy populations or under specific exercise regimens, emphasizing the importance of individual variability and contextual moderators.

### 5.4 Oats

Due to its high soluble fiber content and β-glucans, oat bran has demonstrated immunomodulatory and anti-inflammatory properties, particularly in populations with metabolic risk factors (So et al., 2024). While yielding inconsistent or context-dependent outcomes, the combined effects of oat bran and exercise on immune function are particularly intriguing (Table 3). In a clinical study by Abidin et al. (2024), 6 weeks of oat bran supplementation (18 g/day) in hypercholesterolemic women led to significant increases in eosinophil and neutrophil counts, suggesting improved innate immunity. However, when combined with moderate aerobic activity (brisk walking, 3×/week), there was a surprising reduction in cytotoxic T cells (CD8^+^) and NK cells (CD16^+^). This finding suggests a potentially non-additive or even counterbalancing interaction, possibly due to immune cell redistribution or adaptation in response to concurrent stimuli. Further complexity was revealed by Davis et al. (2004), who used a murine model of HSV-1 respiratory infection. Both oat β-glucan supplementation and moderate treadmill exercise independently enhanced immune defense—β-glucans improved macrophage function, and exercise increased NK cell activity and reduced mortality. However, their combination did not yield additional protective effects, indicating that specific immune mechanisms may reach a ceiling effect when stimulated via parallel pathways. Contrastingly, Dong et al. (2024) reported a synergistic benefit in a high-fat diet (HFD) model-induced skeletal muscle dysfunction. Oat bran combined with moderate-intensity exercise reduced oxidative stress markers (SOD, GSH) and pro-inflammatory cytokines (TNF-α, IL-1β, IL-6), while also enhancing grip strength and improving both carbohydrate and lipid substrate metabolism during exercise. This promising intervention modulated gut microbiota in favor of SCFA-producing taxa, linking microbial activity with improved muscle performance and reduced inflammation.

**TABLE 3:** Impact of oat bran and exercise on immunity and inflammation.

Natural products	Dose	Exercise	Exercise protocol	Disease/Condition	Mechanism	Synergistic/Additive	Ref.
Oat Bran	18 g/day	Brisk walking	30 min/session, 3×/week, 6 weeks	hypercholesterolemia women	↑ Eosinophils, Neutrophils (Ob group); ↓ CD8^+^, CD16^+^ cells (ObEx group)	Unclear/Potentially conflicting	Abidin et al. (2024)
Oat Beta-Glucan	In drinking water, 10 days	Treadmill	1 h/day, 6 days	HSV-1 respiratory infection	↑ Macrophage resistance (β-glucan and exercise), ↑ NK cell activity (exercise only), ↓morbidity/mortality (exercise)	No effect	Davis et al. (2004)
Oat Bran	200 g/1,044.4 g feed, 8 weeks	Moderate intensity	3 days/week for 8 weeks	HFD-induced muscle dysfunction	↓TNF-α, IL-1β, IL-6, SOD, GSH; ↑ grip strength and endurance; regulated muscle-related gene expression; ↑ exercise-induced carbohydrate and lipid metabolism; ↑ SCFA-producing gut microbes; ↓inflammatory metabolites	Synergistic	Dong et al. (2024)

### 5.5 Mango

Mango leaf extract is increasingly recognized for its anti-inflammatory and antioxidant capabilities, primarily attributed to its rich mangiferin content and other phenolic compounds (Kumar et al., 2021; Kim et al., 2021). These bioactives reduce chronic inflammation by mitigating oxidative stress and downregulating pro-inflammatory cytokines such as IL-12 and TNF-α (Mohammaddoost et al., 2024a; Mohammaddoost et al., 2024b). This mechanism is especially beneficial in obese populations, where persistent low-grade inflammation is a hallmark feature. Recent studies have begun to explore the combined effects of mango leaf extract supplementation and structured exercise interventions. In a clinical trial by Mohammaddoost et al. (2024b), Overweight young men (aged 20–25) were assigned to receive either TRX training (3×/week for 6 weeks), 1,000 mg/day mango leaf extract, or a combination of both. The group receiving the combined intervention experienced greater TNF-α, BMI, and waist-to-hip ratio reductions than either intervention alone, indicating an additive or potentially synergistic effect on inflammation and adiposity (Table 4). A separate study (Mohammaddoost et al., 2024a) corroborated these findings, demonstrating that mango leaf supplementation (500 mg, twice daily) combined with TRX training significantly reduced IL-12, another key cytokine associated with chronic inflammation. These benefits extended beyond inflammation to include significant improvements in body composition, such as reduced BMI and WHR, offering hope for the future of metabolic health. Mechanistically, TRX resistance training is known to independently influence cytokine profiles by enhancing anti-inflammatory responses through muscle-derived myokines and improved metabolic function (Cerqueira et al., 2020; Lee and Lee, 2021).

**TABLE 4:** Impact of mango and exercise on immunity and inflammation.

Natural products	Dose	Exercise	Exercise protocol	Disease/Condition	Mechanism	Synergistic/Additive	Ref.
Mango	1,000 mg/day	TRX resistance training	6 weeks, 3 sessions/week	Obesity-related inflammation	↓ TNF-α, ↓ BMI, ↓ WHR, especially in exercise + extract group	Additive	Mohammaddoost et al. (2024b)
Mango	1,000 mg/day	TRX resistance training	6 weeks, 3×/week	Obesity-related inflammation	↓ TNF-α, ↓ BMI, ↓ WHR; Mango leaf + TRX > mango alone or control	Additive	Mohammaddoost et al. (2024b)

However, exercise alone—especially in moderate to high intensity—can also trigger oxidative stress without adequate antioxidant capacity. The antioxidant activity of mango leaf extract provides a reassuring buffering effect, augmenting the exercise-induced anti-inflammatory benefits by reducing ROS and modulating immune signaling pathways (Mohammaddoost et al., 2024b). These studies suggest combining plant-based bioactives with functional exercise modalities like TRX can produce superior outcomes in metabolically at-risk individuals. The dual action on inflammatory cytokine suppression and body composition regulation supports mango leaf extract as a valuable adjunct in lifestyle-based interventions targeting obesity and related inflammatory conditions.

### 5.6 Pomegranate

Pomegranate (Punica granatum) extract, due to its high levels of polyphenols—notably punicalagin and ellagic acid—exerts significant anti-inflammatory and antioxidant effects, particularly in populations with type 2 diabetes (T2D), obesity, and postmenopausal women, who are prone to oxidative stress and immune dysregulation (Cordiano et al., 2024; Wang et al., 2020; Siddiqui et al., 2024; Ammar et al., 2017; Morvaridzadeh et al., 2020). These bioactives have been shown to downregulate key inflammatory markers, such as C-reactive protein (CRP), IL-6, and TNF-α, while improving systemic total antioxidant capacity (TAC). Exercise alone—both aerobic and resistance training—is known to upregulate antioxidant enzyme systems (SOD, GSH, glutathione peroxidase (GPx)) and reduce inflammation through modulation of cytokine profiles and improvements in insulin sensitivity (Simioni et al., 2018; Sirico et al., 2018; Kirwan et al., 2017). However, the role of pomegranate supplementation in reducing oxidative stress is even more promising. In a randomized clinical trial on postmenopausal women with T2D (Yarmohammadi and Mahjoub, 2017), those receiving both pomegranate juice (150 mL/day) and aerobic training (3×/week) showed significantly higher increases in SOD, GPx, GSH, and TAC than groups receiving either intervention alone. This indicates synergistic antioxidant effects, critical in reducing diabetes-related oxidative damage.

Similarly, Akbarpour et al. (2024) demonstrated that combining 200 mg/day pomegranate capsules with intensive resistance training (5×/week) markedly reduced CRP, CK, and LDH levels—biomarkers of muscle damage and inflammation—compared to placebo or single interventions, particularly for CRP, which showed a −36.6% decrease. In another study, hydroethanolic pomegranate peel extract combined with HIIT increased SOD and catalase (CAT), while reducing CRP in models of exercise-induced oxidative stress (Nameni and Aliakbar Alavi, 2021).

In diabetic populations, the combination of pomegranate juice and resistance or aerobic exercise led to greater improvements in lipid profiles (↑ increased high-density lipoprotein (HDL), ↓ decreased low-density lipoprotein (LDL)), adiponectin levels, and inflammatory cytokines (↓ decreased interleukin-6 (IL-6), TNF-α) than either treatment alone (Jahed et al., 2022; Abdi, 2018; Akbarpour et al., 2022).

Notably, outcomes like increased TAC and reduced malondialdehyde (MDA) were only observed in combination groups, suggesting that pomegranate’s effectiveness is enhanced by exercise stimuli (Bonab, 2020). Mechanistically, this synergism may stem from enhanced exercise-induced reactive oxygen species (ROS) scavenging by polyphenols. Amplified anti-inflammatory cytokine responses (e.g., ↑ increased IL-10). Improved mitochondrial function and insulin signaling in metabolic tissues. These findings position pomegranate extract as a powerful adjunct to structured physical activity, especially in populations vulnerable to oxidative damage and chronic inflammation, such as those with T2D, obesity, or age-related metabolic decline (Table 5).

**TABLE 5:** Impact of pomegranate and exercise on immunity and inflammation.

Natural products	Dose	Exercise	Exercise protocol	Disease/Condition	Mechanism	Synergistic/Additive	Ref.
Pomegranate extract	150 mL/day	Aerobic exercise	6 weeks, 3×/week, 45+ min/session	Type 2 diabetes and oxidative stress	↑ GPX, ↑ SOD, ↑ GSH, ↑ TAC; combo (exercise + extract) group had greatest antioxidant improvements	Additive/Synergistic	Yarmohammadi and Mahjoub (2017)
Pomegranate capsule	200 mg/day	Resistance training	8 weeks, 5×/week, 60%–80% 1RM, pyramid method	Muscle damage and inflammation	↓ CRP (36.56%), ↓ CK (33.44%), ↓ LDH (7.82%) in supplement group (all p < 0.01); ↓ more than placebo; only CRP diff.	Additive/Synergistic (CRP)	Akbarpour et al. (2024)
Pomegranate juice (Punica granatum L.)	150 mL/day, 6 weeks	Aerobic training	6 weeks, 3×/week, 25–45 min/session, 60%–75% HRR	Type 2 Diabetes, insulin resistance	↑ Adiponectin, ↓ Insulin Resistance (both significant); no sig. change in Resistin among groups	Additive	Abdi (2018)
Pomegranate juice	100 mL/day, 8 weeks	Resistance training	3×/week, 30%→80% 1-RM progressive load	Type 2 Diabetes, inflammation	↑ IL-10, ↑ HDL; ↓ CRP, IL-6, TNF-α, LDL	Synergistic	Akbarpour et al. (2022)
pomegranate peel extract	1 mL of pomegranate/day, 8 weeks	HIIT	8 weeks	Exercise-induced oxidative stress	↑ SOD, ↑ CAT, ↓ CRP	Synergistic	Nameni and Aliakbar Alavi (2021)
Pomegranate extract	225 mg/day	Resistance training	28 sessions of 90 min over 4 weeks	Obesity/Oxidative Stress	↓ MDA; ↑ TAC (only in combo group)	Synergistic	Jahed et al. (2022)

### 5.7 Dark chocolate

Dark chocolate, particularly with high cocoa content (≥70–80%), is rich in flavonoids such as epicatechin, catechin, and procyanidins, which possess robust antioxidant and anti-inflammatory properties (Samanta et al., 2022; Behzadi et al., 2024).

These bioactives mitigate oxidative stress by neutralizing ROS and downregulating pro-inflammatory cytokines such as TNF-α, IL-6, and hs-CRP. Additionally, they improve adipokine profiles, enhancing adiponectin levels while reducing leptin, resistin, and monocyte chemoattractant protein-1 (MCP-1)—biomarkers often dysregulated in obesity and metabolic syndrome (Samanta et al., 2022).

On the other hand, moderate-intensity exercise, including interval jump rope exercise (JRE) and circuit training, has been shown to improve immune surveillance and metabolic homeostasis. These exercises enhance endogenous antioxidant defenses (↑ SOD, GPx, TAC), reduce fat mass and waist circumference, and increase activity of innate and adaptive immune cells such as neutrophils, T-helper cells, and lymphocytes (Eskandari et al., 2020; Hooshmand Moghadam et al., 2021; Grosso et al., 2022). However, it is important to note that excessive or high-volume exercise may induce oxidative damage, immune suppression, and increase injury risk, underscoring the importance of dosing exercise appropriately. High-volume exercise can lead to overtraining syndrome, resulting in decreased performance, increased risk of injury, and immune system suppression (Razavi Majd and Ghahramani, 2019). Emerging evidence supports a synergistic interaction between dark chocolate supplementation and structured exercise in improving immunometabolic health (Table 6). This means that when dark chocolate and exercise are combined, their individual effects are amplified, resulting in greater health benefits than if used separately. In obese adolescent boys, 30 g/day of 83% dark chocolate plus JRE (3×/week for 6 weeks) resulted in significantly elevated antioxidant markers (↑ SOD, GPx, TAC) and reduced lipid peroxidation (↓ TBARS), beyond the effects of either intervention alone (Hooshmand Moghadam et al., 2021).

**TABLE 6:** Impact of dark chocolate and exercise on immunity and inflammation.

Natural products	Dose	Exercise	Exercise protocol	Disease/Condition	Mechanism	Synergistic/Additive	Ref.
Dark chocolate (83% cocoa)	30 g/day	Jump rope exercise	3 sessions/week,6 weeks	Obesity/inflammation	↓ TNF-α, IL-6, hs-CRP, leptin, resistin, RBP-4, MCP-1, chemerin; ↑ adiponectin, irisin; ↓ body fat, waist-hip ratio	Synergistic	Eskandari et al. (2020)
Dark chocolate (83% cocoa)	30 g/day	Jump rope exercise	3 sessions/week for 6 weeks	Obesity/Oxidative Stress	↑ TAC, SOD, GPx; ↓ TBARS	Synergistic	Hooshmand Moghadam et al. (2021)
Chocolate malt drink	45 g malt powder/day in 300 mL water	Circuit training	3 sessions/week, 6 weeks; supplement 30 min pre-exercise	Immune function	↑ WBC and neutrophils, ↑ T helper cells, ↑ lymphocytes and subsets	Additive	Liew et al. (2013)

Furthermore, Eskandari et al. (2020) demonstrated that the same intervention significantly downregulated inflammatory markers (TNF-α, IL-6, hs-CRP) and adipokines (leptin, resistin, chemerin, MCP-1), while increasing adiponectin and irisin, suggesting potent anti-inflammatory and metabolic synergy. In another study, circuit training combined with a flavonoid-rich chocolate malt beverage (45 g malt powder in 300 mL water, consumed 30 min pre-exercise) significantly enhanced white blood cell (WBC) counts, T-helper cell proliferation, and lymphocyte subsets, indicating improvements in both innate and adaptive immunity (Liew et al., 2013). These findings suggest that dark chocolate flavonoids may augment exercise-induced physiological adaptations by enhancing endogenous antioxidant enzyme systems, modulating cytokine signaling, improving adipokine balance, and immune cell activation. However, the degree of synergy appears to be context-dependent, influenced by factors such as Age, sex, and baseline metabolic state, Training modality, and exercise intensity, Flavonoid bioavailability, which varies with gut microbiota composition and genetic polymorphisms (Decroix et al., 2018). Most current studies have been conducted on male adolescents with obesity, which limits the generalizability to female, elderly, or lean individuals. Therefore, future studies should consider diverse populations and explore dose–response relationships for cocoa flavonoids and exercise. This emphasis on the importance of understanding the complexities of the research should make readers feel included and considered in the ongoing scientific exploration.

### 5.8 Ginseng

Panax ginseng, a well-documented adaptogenic herb, is a testament to the potential of natural remedies in healthcare. Its potent immunomodulatory and antioxidant properties, primarily attributed to its rich composition of ginsenosides, phenolic compounds, and saponins (Ratan et al., 2021; Park et al., 2021; Hyun et al., 2022), inspire us to explore the possibilities of herbal medicine. Mechanistically, ginseng enhances NK cell activity, regulates cytokine production, and modulates innate and adaptive immunity. It has also been shown to attenuate oxidative stress in tissues vulnerable to inflammatory damage, particularly the cardiac muscle, which is clinically relevant in infectious or inflammatory cardiovascular diseases (Athari et al., 2022). Ginseng supplementation stimulates endogenous antioxidant defenses through the upregulation of SOD, GPx, and TAC. Additionally, it exerts anti-inflammatory effects by suppressing signaling pathways such as NF-κB and MAPK, thereby reducing the expression of key pro-inflammatory cytokines, including TNF-α, IL-1β, IL-6, and IL-8 (Park et al., 2021; Hyun et al., 2022; Athari et al., 2022).

In parallel, aerobic exercise, particularly at moderate intensity, contributes significantly to immune homeostasis by enhancing mitochondrial efficiency, promoting vascular perfusion, and stimulating anti-inflammatory myokines (Abd El-Kader and Al-Shreef, 2018; Wiecek et al., 2018; Da Silveira et al., 2021). It downregulates systemic inflammation by reducing the expression of pro-inflammatory cytokines and improving immune surveillance, particularly under chronic or infection-driven inflammatory conditions. A pivotal preclinical study by Athari et al. (2022) investigated the interactive effects of ginseng supplementation (0.025 mg/kg/day, IP) and aerobic exercise (60% VO_2_max, 5 days/week) in rats with *Listeria* monocytogenes-induced infective endocarditis. After 4 weeks, the combined intervention led to significantly greater increases in TAC, SOD, GPx, and paraoxonase-1 (PON-1), as well as more substantial reductions in TNF-α, IL-1, IL-6, and IL-8 levels in cardiac tissue, compared to either intervention alone. These findings indicate a synergistic anti-inflammatory and antioxidant effect, likely driven by the convergence of ginseng’s molecular modulation of oxidative and inflammatory signaling with exercise-induced systemic improvements in immune regulation and oxidative metabolism (Table 7). Mechanistically, this synergy arises from Exercise-induced improvements in circulation, oxygen delivery, and myokine-mediated inflammation resolution. Ginseng-mediated inhibition of oxidative stress and transcriptional suppression of inflammatory cytokines via NF-κB blockade. Together, these complementary pathways amplify reductions in cardiac inflammation and oxidative damage, particularly under infectious inflammatory stress, inspiring us about the potential of natural remedies in healthcare.

**TABLE 7:** Impact of ginseng and exercise on immunity and inflammation.

Natural products	Dose	Exercise	Exercise protocol	Disease/Condition	Mechanism	Synergistic/Additive	Ref.
Ginseng	0.025 mg/kg (IP) daily	Aerobic exercise	5 days/week, 4 weeks, 60% VO_2_ max	*Listeria* monocytogenes-induced endocarditis	↑ TAC, SOD, GPx, PON-1; ↓ IL-1, IL-6, IL-8, TNF-α in cardiac tissue	Synergistic	Athari et al. (2022)

### 5.9 Green tea

Green tea, particularly due to its high epigallocatechin gallate (EGCG) concentration, exhibits substantial antioxidant, anti-inflammatory, and cardioprotective properties. These polyphenolic compounds modulate the immune system by downregulating pro-inflammatory cytokines such as TNF-α and IL-6 while upregulating anti-inflammatory mediators including IL-10 (Golpasandi et al., 2024; Alikhani et al., 2021; Vakili and Hosseinpour, 2015). Additionally, EGCG influences redox-sensitive signaling pathways, particularly NF-κB, thereby mitigating oxidative stress and supporting endothelial and metabolic function, especially in obese and aging populations (Saed-mocheshi et al., 2020; Fathei et al., 2016; Zhang et al., 2020; Naghizadeh and Hemati Farsani, 2023).

In parallel, aerobic and HIIT independently enhance systemic antioxidant defenses (e.g., SOD, GPx, TAC) and modulate inflammation via myokine secretion (e.g., irisin, adiponectin), improved mitochondrial function, and vascular health (Zhu et al., 2021; Rohnejad and Monazzami, 2023).

Several studies have investigated the interactive or synergistic effects of green tea supplementation and structured exercise (Table 8). Bagheri et al. (2020) reported that 500 mg/day of green tea extract combined with moderate endurance training significantly improved metabolic and inflammatory markers (↓ IL-6, ↓ hs-CRP, ↑ adiponectin, ↑ irisin), with effects greater than either intervention alone. In patients with type 2 diabetes, Golpasandi et al. (2024) found that green tea combined with HIIT led to greater reductions in NT-proBNP and GDF-15, indicating improved cardiac stress response. Vakili and Hosseinpour (2015) and Naghizadeh and Hemati Farsani (2023) demonstrated significant synergistic effects on antioxidant enzyme activity (e.g., ↑ TrxR-1, ↑ PON-1) and suppression of IL-1β and IL-6. However, not all studies support synergy. Alikhani et al. (2021) observed reductions in IL-10 and TNF-α with green tea and spinning exercise, but without additive effects on body composition. Yang et al. (2011) and Saed-mocheshi et al. (2020) reported that although green tea suppressed exercise-induced NF-κB activation, broader metabolic benefits were not enhanced. Arabzadeh et al. (2022), did not observe any synergistic effect between green coffee and treadmill training on apoptotic signaling in cardiac tissue. The outcome variability highlights the context-specific nature of green tea–exercise interactions. Key moderating factors include exercise type and intensity (e.g., HIIT vs. aerobic vs. resistance), polyphenol dosage and form (extract, brewed, capsule), population characteristics (e.g., age, metabolic health), and targeted biomarkers (inflammation, metabolism, apoptosis). Notably, synergistic effects are more pronounced in metabolically dysregulated individuals, where baseline inflammation and oxidative stress are elevated and thus more modifiable by intervention (Golpasandi et al., 2024; Vakili and Hosseinpour, 2015; Bagheri et al., 2020). There is a possibility of synergy resulting from the combined inhibition of NF-κB signaling (Zhang et al., 2020), an increased antioxidant capacity facilitated by both external polyphenols and the upregulation of internal enzymes, and the modulation of myokines (such as irisin and adiponectin) that enhance immune and metabolic responses (Naghizadeh and Hemati Farsani, 2023). These mechanisms highlight that while green tea and exercise are independently beneficial, their combination is most effective in targeted, at-risk populations when appropriately matched in dose, duration, and intensity.

**TABLE 8:** Impact of green tea and exercise on immunity and inflammation.

Natural products	Dose	Exercise	Exercise protocol	Disease/Condition	Mechanism	Synergistic/Additive	Ref.
Green coffee	300 mg/kg, 5 days/week, 12 weeks	Treadmill	17–27 m/min, 60%–75% VO_2_peak, 5 days/week, 12 weeks	Cardiac apoptosis/myocardial oxidative stress	↓ HIF-1α, ↓ BNIP3, ↓ IGFBP3, ↓ Bax, caspase-3; ↑ Bcl-2, ↑ GPx; ↓ MDA; ↑ Bax/Bcl-2 ratio	No effect	Arabzadeh et al. (2022)
Green tea	500 mg/day for 8 weeks	Endurance training	3x/week; moderate intensity, 40%–59% HRR, 8 weeks	Overweight/Inflammation/metabolic syndrome	↑ Irisin, ↑ Adiponectin, ↓ IL-6, ↓ hs-CRP; ↔TNF-α; ↓ weight, BMI, body fat, VFA	Synergistic	Bagheri et al. (2020)
Green coffee	>5 cups/day	Aerobic exercise	HRmax 60%–70%, 60 min/day,12 weeks	Obesity	↓ TNF-α, ↓ IL-6, ↓ Leptin; ↔body weight; ↓ waist-to-hip ratio	No effect	Yang et al. (2011)
Green tea	800 mg of green tea/daily for 8 weeks	HIIT	8 weeks, 3×/week, 6 × 1-min bouts at 90%–95% MHR + 4-min rests at 70%–75% MHR	Obesity/Type 2 diabetes	↓ GDF-15, ↓ NT-proBNP, ↓ serum glucose, ↓ insulin resistance	Synergistic	Golpasandi et al. (2024)
Green tea	450 mg three times daily for 8 weeks	Spinning	3x/week, RPE 11–17,8 weeks	Overweight	↓ BMI, ↓ BF%, ↓ TNF-α; ↑ IL-10	No effect	Alikhani et al. (2021)
Green tea	2.5 g green tea 3 times/daily for 8 weeks	Aerobic training	60 min @ 55%–65% HRR, 3x/week,8 weeks	Obesity	↓ hs-CRP, ↓ LDL-C, ↑ HDL-C, ↓ subcutaneous fat; ↔TG	Synergistic	Vakili and Hosseinpour (2015)
Green tea	1.3 mL of 10 mg/100 mL solution, 3×/week via gavage	Treadmill	3 sets of 15 min, 3–10 m/min, 5x/week	Prostate inflammation/cancer prevention	↑ NF-κB; ↓ NF-κB; ↔ COX-2 or p53	Modulatory	Saed-Mocheshi et al. (2020)
Green tea	3x/day	Aerobic	45–60 min/session, 3x/week,8 weeks	Inactive/obesity	↓ TC, TG, LDL; ↑ HDL; ↓ CRP; ↔ HDL	Additive	Fathei et al. (2016)
Yunkang 10 green tea (YKGT)	Unspecified extract dose (high in EGCG, caffeine)	Treadmill	8 weeks	Metabolic Syndrome	↓ Glucose, insulin, TC, TG, ALT; ↓ NF-κB signaling; ↓ lipid synthesis genes; ↑ glucose transport in muscle	Additive	Zhang et al. (2020)
Green tea	450 mg/day	HIIT	3×/week, 90% HRR,8 weeks	Inflammation/oxidative stress	↑ TrxR-1, ↑ PON-1; ↓ IL-1β, ↓ IL-6, ↓ galanin	Synergistic	Naghizadeh and Hemati Farsani (2023)
Green tea	500 mg/day,12 weeks	Aqua training	12 weeks, 3×/week, 60 min/session, intensity: 65%–75% HR	Inactive/obesity	↓ TNF-α, ↓ CRP	Synergistic	Bonab (2020)

### 5.10 Garlic

Garlic—particularly in the form of Aged Garlic Extract (AGE)—contains bioactive sulfur compounds such as S-allylcysteine (SAC), which exhibit powerful anti-inflammatory and antioxidant effects. These effects are primarily mediated by inhibiting the NF-κB and TLR4 signaling pathways (Ghyasi et al., 2019; Khatami et al., 2020; Sadeghpour Firozabadi et al., 2021; Hosseinzadeh et al., 2023). Garlic supplementation has been shown to reduce pro-inflammatory cytokines, including TNF-α, IL-6, and Fetuin-A, while enhancing NK cell activity and promoting a more balanced cytokine profile, supporting both innate and adaptive immune responses (Mohammadi Sarableh et al., 2022; Firozabadi et al., 2023).

Furthermore, the sulfur compounds in garlic act as free radical scavengers and inhibit oxidative stress pathways (Sánchez-Gloria et al., 2022). Parallel to this, aerobic exercise independently contributes to reducing systemic inflammation—especially in obese or metabolically impaired individuals—by lowering levels of hs-CRP, TNF-α, and IL-6, enhancing insulin sensitivity, and boosting the activity of antioxidant enzymes such as SOD and GPx (Hassanen et al., 2024; Farazandeh Nia et al., 2018; Khosravi et al., 2024; Khabiri et al., 2022). Exercise also decreases lipid peroxidation markers like MDA and increases anti-inflammatory cytokines (e.g., IL-10), while lowering Lipopolysaccharides (LPS) levels, thereby modulating immune responses (Soori et al., 2015; Enayatjazi et al., 2022).

Emerging evidence suggests that combining garlic supplementation and aerobic or resistance exercise results in significantly greater improvements in inflammatory and oxidative stress markers than either intervention alone (Table 9). Studies in both animals and humans demonstrate reductions in NF-κB, TLR4, IL-6, TNF-α, and hs-CRP, alongside increases in SOD, GPx, and IL-10 with combined interventions (Razavi Majd and Ghahramani, 2019; Khatami et al., 2020; Sadeghpour Firozabadi et al., 2021; Hosseinzadeh et al., 2023; Mohammadi Sarableh et al., 2022; Farazandeh Nia et al., 2018; Khabiri et al., 2022; Enayatjazi et al., 2022; Akbarpour and Ghobadipour, 2019; Moosavian et al., 2020; Zhao et al., 2024; Khabiri et al., 2023; Jamadi et al., 2023; Gholami et al., 2020). For example, Khabiri et al. (2023) showed that obese rats receiving 600 mg/kg/day of AGE along with aerobic training exhibited significant decreases in Fetuin-A, NF-κB, and TLR4 in liver and adipose tissue, along with improvements in insulin resistance (HOMA-IR), surpassing the effects of garlic or exercise alone. Similarly, Jamadi et al. (Jamadi et al., 2023) garlic and aerobic exercise enhanced antioxidant enzyme activity and normalized hormone levels more effectively than single treatments in rats exposed to electromagnetic radiation. In a clinical study, Gholami et al. (2020) reported that overweight men taking 1,000 mg/day of garlic combined with aerobic training showed significantly greater reductions in TNF-α and hs-CRP than in control groups. Additional studies have echoed these findings, demonstrating synergistic improvements in body composition, blood pressure, cytokine regulation, and immune function (Khatami et al., 2020; Sadeghpour Firozabadi et al., 2021; Mohammadi Sarableh et al., 2022; Gholami et al., 2020). The synergistic effects likely arise from converging mechanisms. Garlic inhibits inflammatory gene expression by suppressing TLR4/NF-κB pathways, while exercise promotes systemic anti-inflammatory and antioxidant responses. This combination enhances mitochondrial function, improves vascular health, and supports hormonal balance, creating a powerful interplay between dietary and physical activity interventions (Sadeghpour Firozabadi et al., 2021; Khabiri et al., 2022; Khabiri et al., 2023). However, some studies report additive rather than synergistic effects, or show limited impact on specific markers. These inconsistencies may arise from dosage variations, garlic form, exercise modality, duration, and study populations. Thus, further well-controlled trials are needed to define optimal protocols for maximizing synergistic outcomes. In conclusion, the combination of garlic supplementation and regular exercise represents a promising and cost-effective lifestyle intervention to combat chronic inflammation, oxidative stress, insulin resistance, and related metabolic disorders, especially in obese or high-risk populations.

**TABLE 9:** Impact of garlic and exercise on immunity and inflammation.

Natural products	Dose	Exercise	Exercise protocol	Disease/Condition	Mechanism	Synergistic/Additive	Ref.
Aged Garlic Extract (AGE)	600 mg/kg/day	Aerobic Training	5 days/week, 8 weeks	HFD-induced obesity	↓ Body weight, ↓ plasma Fetuin-A, ↓ HOMA-IR, ↓ NF-κB, ↓ TLR4	Synergistic	Khabiri et al. (2023)
Garlic	800 mg/kg/day	Aerobic (Endurance)	3×/week, 60 min/session, 50%–60% max speed, 8 weeks	Rats Under Wi-Fi Electromagnetic Radiation	↑ SOD and GPx, ↓ MDA and FSH	Synergistic	Jamadi et al. (2023)
Garlic	1,000 mg/day	Walking/Running	3×/week, 20–45 min/session at 60%–75% max heart rate, for 8 weeks	Sedentary overweight/chronic inflammation	↓ hs-CRP and ↓ TNF-α	Synergistic	Gholami et al. (2020)
Garlic Supplement	1,000 mg/day	Aerobic training	3 sessions/week, 8 weeks; 55%–65% HR reserve, 30–55 min/session	Obesity/High Blood Pressure	↓ Lcn-2, ↓ IL-1β, ↓ weight, ↓ BMI, ↓ SBP	Synergistic	Khatami et al. (2020)
Garlic	1 mL of garlic extract/100 g BW/day	Resistance training	5 days/week,8 weeks	Diabetic	↑ CTRP3 expression, ↓ IL-6, ↓ HOMA-IR	Synergistic	Sadeghpour Firozabadi et al. (2021)
Garlic homogenate	250 mg/kg	Voluntary exercise	24 h/day,6 weeks	Type 1 diabetic	↓ HbA1c, ↓ cholesterol, ↑ TAC, ↓ MDA; improved cardiac histology (↓ edema, leukocyte infiltration, necrosis)	Additive	Ghyasi et al. (2019)
Garlic	500 mg/kg/day	Aerobic Training	5 sessions/week, 15–48 min per session, speed 10–24 m/min, for 8 weeks	Parkinson’s Disease	↑ SOD, ↑ GPx, ↓ MDA	Additive	Hosseinzadeh et al. (2023)
Garlic	1,000 mg/day	Progressive Resistance Training	8 weeks, 3×/week, 3 sets × 10 reps @ 10RM, 1-min rest	Overweight women/Inflammation/insulin resistance	↓ hs-CRP, ↓ HOMA-IR, ↓ glucose, ↑ lean mass, ↓ fat mass	Synergistic	Mohammadi Sarableh et al. (2022)
Garlic	1 mL/100 g BW/day	Treadmill	8 weeks, 5 days/week, 10–18 m/min, 10–40 min/session	Diabetic	↓ IL-17, ↓ IL-22, ↓ HOMA-IR	Synergistic	Sadeghpour Firozabadi et al. (2023)
Garlic	1 mL/kg/BW	Swimming	8 weeks, 3 sessions/week, 60 min/session	Obesity	↑ IL-10, ↓ TNF-α	Additive	Farazandeh Nia et al. (2018)
Garlic	500 mg/kg/day	Aerobic Training	8 weeks, 5 sessions/week, 15–48 min/session, speed 10–24 m/min	Parkinson’s Disease	↓ IFN-γ, ↑ IL-4, ↑ motor balance	Synergistic	Khosravi et al. (2024)
Garlic	250 mg/day	Combined (Endurance + Resistance)	8 weeks, endurance: 60%–80% HRmax running; resistance: 40%–85% 1RM weight training	Oxidative stress/antioxidant defense	↓ MDA, ↑ TAC	Additive or possibly synergistic effects	Khoobkhahi et al. (2019)
Aged Garlic Extract (AGE)	600 mg/kg/day	Aerobic training	5 days/week, for 8 weeks	Obesity/inflammation/dyslipidemia	↓ TLR4, ↓ NF-κB, ↑ lipid profile	Synergistic	Khabiri et al. (2022)
Garlic	2tablets/day,10 weeks	Concurrent (Aerobic + Resistance)	70%–80% HRmax aerobic + resistance training, 10 weeks	Obesity/Insulin Resistance	↓ Lipocalin-2, ↓ insulin, ↓ HOMA-IR	Synergistic	Soori et al. (2015)
Garlic	1 mL/kg/day	Endurance ()	5x/week,8 weeks	Breast Cancer and inflammation	↓ IL-6, ↓ IL-8, ↓ IL-17, ↑ IL-10	Synergistic	Enayatjazi et al. (2022)
Garlic extract	2.5 g/kg/day	Swimming	8 weeks, 3x/week, 30 min/session	Oxidative stress and inflammation in CKD	↑ SOD, ↑ CAT, ↑ IL-10, ↓ TNF-α, ↓ MDA	Synergistic	Razavi Majd and Ghahramani (2019)
Garlic-Lemon	30 cc/daily	Aerobic	6 weeks, 3x/week, 60%–74% max HR	Obesity-related inflammation/CVD risk	↓ CRP (27%), ↓ fibrinogen (38%), ↓ body fat %	Synergistic	Akbarpour and Ghobadipour (2019)

### 5.11 Spirulina

Spirulina, a nutrient-dense cyanobacterium rich in high-quality proteins, phycocyanin, essential fatty acids, vitamins, and minerals, has demonstrated potent antioxidant and immunomodulatory effects (Brito et al., 2020; Izadmehr et al., 2022). It modulates inflammation primarily through inhibition of the NF-κB signaling pathway and suppression of pro-inflammatory mediators such as TNF-α, IL-6, and CRP, while also enhancing the activity of antioxidant enzymes including SOD and GPx (Oz and Gokbel, 2023; Juszkiewicz et al., 2018; Marcinko et al., 2015; Zunner et al., 2022; Nobari et al., 2022; Calella et al., 2022). Meanwhile, regular physical activity—including resistance training, aerobic exercise, and HIIT—is well known to improve immune surveillance, modulate cytokine profiles, and reduce systemic inflammation through upregulation of anti-inflammatory mediators like IL-10 and mobilization of immune cells (Ammar et al., 2017; Nieman and Wentz, 2019; Khodadadi et al., 2022). A growing body of evidence suggests that spirulina supplementation and structured exercise can elicit synergistic or additive effects on immune and inflammatory parameters. However, the distinction between “synergistic” and “additive” effects is not always strictly upheld in the literature, and some studies broadly refer to interactions as synergistic without direct evidence of interaction beyond additive responses. The following summary aims to apply these terms more precisely, supported by the cited studies. Several studies provide evidence of synergistic effects, where the combination of spirulina and exercise yielded superior outcomes compared to either intervention alone (Table 10). For instance, Agahi et al. (2022) reported that spirulina plus resistance training enhanced antioxidant defenses (↑ SOD, GPx), reduced oxidative stress markers (↓ MDA, miR-125b, miR-146a), and improved cognitive function in a neurotoxicity model. Similarly, Moradi-Kor et al. (2020) found that the combination of spirulina and voluntary exercise mitigated stress-related behavioral deficits and boosted BDNF levels in adolescent rats. Additionally, in animal models of muscle atrophy and inflammation, spirulina used alongside HIIT or resistance training significantly reduced the expression of TWEAK, Fn14, and atrogin-1 genes (Azarniveh et al., 2022; Zar et al., 2022), suggesting a synergistic effect on muscle regeneration pathways. Sangachin et al. (2022) also demonstrated that spirulina and endurance training synergistically decreased pro-inflammatory cytokines, including IL-6, TNF-α, and TGF-β. Additional studies have confirmed synergistic interactions in reducing oxidative stress markers and improving lipid profiles with swimming exercise (Mazzola et al., 2015), as well as in enhancing antioxidant enzyme activity with strength exercise (Araujo et al., 2020) and resistance training (Izadmehr et al., 2022).

**TABLE 10:** Impact of spirulina and exercise on immunity and inflammation.

Natural products	Dose	Exercise	Exercise protocol	Disease/Condition	Mechanism	Synergistic/Additive	Ref.
Spirulina	100 mg/kg/day	Resistance Training	6 weeks, 5 days/week, 50%–100% of body weight	Stanazolol-induced neurotoxicity	↑ SOD, GPx, TAC; ↓ MDA, miR125b, miR146a; ↑ healthy hippocampal cells; ↑ cognitive function (STL, TDC, RDC, PA %)	Synergistic	Agahi et al. (2022)
Spirulina	200 mg/kg/day	Voluntary exercise	15 days (PND 41–55)	Adolescent stress-induced anxiety, depression, oxidative stress, BDNF and 5HT3 receptor changes	↓ MDA, ↑ GPx, ↑ SOD, ↑ BDNF, ↓ 5HT-3R expression	Synergistic	Moradi-Kor et al. (2020)
Spirulina	6 g/day	HIIT	8 weeks; 3 sessions/week; 4–7 reps of 30s run + 30s walk @ 90% max HR	Overweight/obesity/immune modulation	↑ IgA levels, ↓ fat-free mass	Additive	Nobari et al. (2022)
Spirulina	2 g/day, 4 Weeks	HIIT	4 weeks, 3 sessions/week; each session: 10 min warm-up + 25 min HIIT (4-min bouts @ 85%–95% HRR with 3-min active rest @ 50%–70% HRR) + 10 min cool-down; treadmill walking/running	Type 2 Diabetes	↓ MDA; ↔ TAC	Additive	Khodadadi et al. (2022)
Spirulina	26 mg/kg	Swimming	30 min/session, 3x/week, 10 weeks	Oxidative stress/dyslipidemia	↓ TBARS, ↓ cholesterol, ↓ TG	Synergistic	Mazzola et al. (2015)
Spirulina	200 mg/kg/day	Resistance training	5 sessions/week,8 weeks	Muscle adaptation and inflammatory signaling	↑ IL-6, Gp130, JAK, STAT	Synergistic	Zar et al. (2022)
Spirulina	1g/day	Endurance training	1 h/session: treadmill, cycling, stairs at 65% MHR, 3×/week, for 8 weeks	Overweight-related chronic inflammation	↓TGF-β, IL-6, TNF-α	Synergistic	Sangachin et al. (2022)
Spirulina	50 mg/kg BW	HIIT	5 sessions/week for 8 weeks, treadmill at 90% VO_2_max	Obesity/type 2 diabetes/muscle inflammation and atrophy	↓TWEAK, Fn14, and atrogin-1 genes	Synergistic	Azarniveh et al. (2022)
Spirulina	6 g/day	HIIT	8 weeks, 3 sessions/week, 4–7 reps/session of 30 s run + 30 s walk at 90% max HR	Obesity/immune health/fitness	↑ IgA and IgG, ↑ VO_2_max, ↓ BMI, ↓ WHR, ↓ body fat %, ↑ lean mass	Additive	Eyni Gandomani and Reisi (2020)
Spirulina	1.5 g/day	Aerobic training	6 weeks, 60%–72% max heart rate	Type 2 Diabetes/Cardiovascular inflammation	↓ Resistin; ↓ CRP	Additive	Akbarpour and Samari (2020)
Spirulina	50, 150, 500 mg/kg	Strength Exercise	3 days/week for 8 weeks	Intestinal contractility/oxidative stress	↓ Ileum contractile reactivity; ↓ MDA; ↑ antioxidant activity (DPPH assay)	Synergistic	Araujo et al. (2020)
Spirulina	2 × 500 mg tablets/day	Resistance training	8 weeks	Oxidative stress/obesity	↑ SOD, CAT, GSH-Px	Synergistic	Izadmehr et al. (2022)
Spirulina	750 mg/kg/day for 6 weeks	Chronic and exhaustive swimming	Chronic: 1 h/day × 6 weeks; Exhaustive: until fatigue	Oxidative stress/muscle damage/endurance	↓ CK, ↓ MDA, ↑ SOD,CAT	Additive	Oz and Gokbel (2023)
Spirulina	2 × 500 mg/day for 8 weeks	Aerobic exercise	3×/week, 45 min, 60%–65% max HR for 8 weeks	Type 2 diabetes/inflammation	↓ TNF-α, ↓ IL-6, ↓ CRP	Additive	Hooshmand Moghadam et al. (2022)
Spirulina	1,500 mg/day for 6 weeks	Maximal rowing effort	2000-m test on rowing ergometer, pre- and post-supplementation	Immune modulation post-exercise	Modulation of Treg, CTL, NK, and Tδγ lymphocyte populations; ↓ Treg/CTL ratio; preserved anti-infectious immunity	Additive	Juszkiewicz et al. (2018)
Spirulina	50, 150, 500 mg/kg daily, 8 weeks	Strength training	8-week jump protocol with increasing load in water	Oxidative stress/inflammation/muscle damage	↓ CRP, ↓ MDA, ↑ antioxidant capacity (dose-dependent)	Additive	Brito et al. (2020)

In contrast, other studies reflect additive effects, where spirulina and exercise individually contributed to the observed outcomes without clear evidence of interaction. For example, Nobari et al. (2022) reported that spirulina plus HIIT elevated IgA levels in overweight women, indicating enhanced humoral immunity, but also led to a reduction in fat-free mass, with no synergistic gain in body composition. Likewise, in patients with type 2 diabetes, aerobic training and spirulina independently contributed to reductions in CRP, TNF-α, and IL-6, but the combined effects were additive rather than synergistic (Hooshmand Moghadam et al., 2022; Akbarpour and Samari, 2020). Juszkiewicz et al. (2018) also found that spirulina modulated lymphocyte subpopulations post-exercise (e.g., decreased Treg/CTL ratio), helping preserve immune competence, though without a demonstrated interaction beyond individual effects. Additional additive effects were reported in studies evaluating VO_2_max, IgG, BMI, and body fat percentage following spirulina and HIIT co-intervention (Eyni Gandomani and Reisi, 2020) and antioxidant markers after exhaustive swimming (Oz and Gokbel, 2023).

Overall, while many studies report improvements in oxidative stress, immune parameters, and inflammatory cytokines following combined spirulina and exercise interventions, only a subset provides robust evidence for actual synergistic effects. These synergistic outcomes likely arise from the convergence of distinct but complementary mechanisms: Spirulina’s bioactive compounds (e.g., phycocyanin) inhibit oxidative and inflammatory signaling, while exercise induces mitochondrial adaptations, myokine secretion, and immune cell mobilization (Eyni Gandomani and Reisi, 2020; Izadmehr et al., 2022; Juszkiewicz et al., 2018; Agahi et al., 2022; Sangachin et al., 2022). Differences may influence the variability in synergy across studies in subject populations (e.g., obese, diabetic, aged), exercise modalities, spirulina dosage, intervention duration, and outcome domains (e.g., gene expression vs. humoral immunity). In conclusion, the available evidence supports additive and synergistic interactions between spirulina supplementation and exercise. However, to ensure scientific precision, future reviews and experimental studies should rigorously distinguish these interaction types and avoid labeling results as “synergistic” unless statistically supported by interaction analyses. This nuanced interpretation is essential for developing effective integrative interventions targeting inflammation, oxidative stress, and immune dysregulation.

## 6 Discussion

The results are still inconsistent and often contradictory, despite the research suggesting positive synergistic benefits between exercise and several natural products. One important element contributing to this diversity is the lack of stratification based on individual characteristics, such as age, sex, genetic background, baseline inflammation, metabolic state, and gut microbiota composition.

It is crucial to note that variations significantly influence the physiological outcomes of combined interventions in immune responsiveness, metabolic flexibility, and gut permeability. The current research landscape, particularly in preclinical models, often features homogeneous populations, typically young, overweight male subjects. While applicable in some contexts, this approach limits the generalizability of findings to other demographics, such as older adults, females, athletes, and individuals with comorbidities. The lack of diversity in these studies raises questions about the applicability of observed effects to broader populations.

It is important to consider factors such as training status, disease burden, medication use, and hormonal state (e.g., menopausal status) when interpreting results. Moreover, the interaction between individual variability and dose-response relationships, supplement forms (e.g., extract vs. whole food), and exercise timing can significantly influence the effectiveness of interventions. For instance, depending on the sensitivity of immune cell subsets or the degree of oxidative stress, the effects of interventions such as oat bran, ginseng, or pomegranate on immune modulation or inflammation may vary among individuals.

Thus, individual variability must be considered in future research designs. This is not just a suggestion, but a necessity. It includes stratified study designs that compare outcomes across age groups, sexes, and genetic profiles; exploring population-specific responses in populations such as the elderly, athletes, females, and those with metabolic or inflammatory disorders; comprehending the mechanistic basis for inter-individual variability, especially at the level of immune signaling, gene expression, and microbiota-host interactions; and evaluating the long-term sustainability, safety, and dose optimization of natural product–exercise combinations with an emphasis on individual variability.

By integrating these elements into individualized exercise and supplementation plans, future studies can maximize therapeutic results in various clinical contexts and demographics. This change to more individualized approaches can revolutionize the field, improving patient outcomes and the effectiveness of interventions, offering hope for the future.

## 7 Conclusion

The interplay between natural products and exercise plays a crucial role in maintaining immune function and reducing inflammation. This review highlights the significant benefits of moderate physical activity, which enhances immune responses while mitigating chronic inflammatory conditions. Natural products, such as probiotics, omega-3 fatty acids, and antioxidants, complement these effects by providing additional support to the immune system and helping regulate inflammatory pathways. The evidence suggests that a balanced approach incorporating both exercise and specific dietary components can lead to improved health outcomes, particularly for populations at risk for chronic diseases. However, the effectiveness of these interventions can vary based on individual factors, including age, sex, and genetic predispositions. This highlites the need for personalized nutrition and exercise strategies to optimize immune health.
